# Rotational Thromboelastometry in High-Risk Patients on Dual Antithrombotic Therapy After Percutaneous Coronary Intervention

**DOI:** 10.3389/fcvm.2021.788137

**Published:** 2021-12-22

**Authors:** Anne-Marije Hulshof, Renske H. Olie, Minka J. A. Vries, Paul W. M. Verhezen, Paola E. J. van der Meijden, Hugo ten Cate, Yvonne M. C. Henskens

**Affiliations:** ^1^Central Diagnostic Laboratory, Maastricht University Medical Center+, Maastricht, Netherlands; ^2^Department of Biochemistry, Cardiovascular Research Institute Maastricht, Maastricht University, Maastricht, Netherlands; ^3^Department of Internal Medicine, Maastricht University, Maastricht, Netherlands; ^4^Thrombosis Expert Centre Maastricht, Maastricht University Medical Center+, Maastricht, Netherlands

**Keywords:** percutaneous coronary intervention, thromboelastometry (ROTEM®), fibrinolysis, anticoagulants, antiplatelet drug

## Abstract

**Aims:** Patients using antithrombotic drugs after percutaneous coronary intervention (PCI) are at risk for bleeding and recurrent ischemia. We aimed to explore routine and tissue plasminogen activated (tPA) ROTEM results in a post-PCI population on dual antithrombotic treatment.

**Methods and Results:** In this prospective cohort, 440 patients treated with double antithrombotic therapy after recent PCI and with ≥3 risk factors for either ischemic or bleeding complications were included and compared with a control group (*n* = 95) consisting of perioperative patients not using antithrombotic medication. Laboratory assessment, including (tPA) ROTEM, was performed one month post-PCI and bleeding/ischemic complications were collected over a five-month follow-up. Patients were stratified by antithrombotic regimen consisting of a P2Y12 inhibitor with either aspirin (dual antiplatelet therapy; DAPT, *n* = 323), a vitamin K antagonist (VKA, *n* = 69) or a direct oral anticoagulant (DOAC, *n* = 48). All post-PCI patients had elevated ROTEM clot stiffness values, but only the DAPT group additionally presented with a decreased fibrinolytic potential as measured with tPA ROTEM. Patients receiving anticoagulants had prolonged clotting times (CT) when compared to the control and DAPT group; EXTEM and FIBTEM CT could best discriminate between patients (not) using anticoagulants (AUC > 0.97). Furthermore, EXTEM CT was significantly prolonged in DAPT patients with bleeding complications during follow-up (68 [62–70] vs. 62 [57–68], *p* = 0.030).

**Conclusion:** ROTEM CT has high potential for identifying anticoagulants and tPA ROTEM could detect a diminished fibrinolytic potential in patients using DAPT. Furthermore, the ability of EXTEM CT to identify patients at risk for bleeding may be promising and warrants further research.

## Introduction

Patients with coronary artery disease (CAD) undergoing percutaneous coronary intervention (PCI) are generally prescribed dual antiplatelet therapy (DAPT), consisting of aspirin and a P2Y12 inhibitor (P2Y12i), for 6-12 months to prevent recurrent atherothrombotic events (1, 2). In patients with comorbidities, such as atrial fibrillation or a mechanic valve, the P2Y12 inhibitor is often combined with an anticoagulant (3, 4). A delicate balance between limiting ischemic risk while preventing bleeding emerges in patients on antithrombotic treatment. Nowadays, physicians can choose from multiple antithrombotic treatment regimens including the more potent P2Y12 inhibitors prasugrel (5) and ticagrelor (6) next to clopidogrel, and the widespread availability of direct oral anticoagulants (DOACs) in addition to vitamin K antagonists (VKA). However, patients with comorbidities, or with recurring ischemic or bleeding events remain a challenging group that often require individualized treatment strategies. Risk factors for recurrent ischemic and bleeding events show considerable overlap, further complicating prediction and subsequently prevention of these adverse events. International guidelines therefore recommend individual assessment of benefit/risk ratios in these high-risk patients (2, 7). Individual benefit/risk evaluation in the form of monitoring multiple antithrombotic drugs and identifying patients at risk for ischemic and/or bleeding events remains a major challenge in clinical practice. Hemostasis tests could potentially characterize patients with either hemostatic abnormalities predisposing for bleeding events, or with a more prothrombotic phenotype leading to recurrent ischemic events despite antithrombotic therapy.

Hemostasis tests and platelet function tests (PFTs) have multiple limitations when monitoring patients on antithrombotic therapy. First, most laboratory assays developed to monitor antithrombotic drugs assess one specific hemostasis pathway. Clear examples are the direct thrombin inhibitors (dabigatran) and factor (F)Xa-inhibitors (rivaroxaban, apixaban, edoxaban), which concentrations are monitored by using the diluted thrombin time and the anti-Xa assay, respectively (8). Alternatively, residual platelet reactivity in patients on antiplatelet drugs (e.g. P2Y12 inhibitor) can be measured using platelet function assays such as Light Transmission Aggregometry (LTA), Platelet Function Analyzer (PFA), VerifyNow and Multiplate (9). Though high and low on-treatment platelet reactivity are associated with recurrent ischemia and bleeding, respectively, clinical risk prediction and subsequent treatment modification in a real-life setting remains suboptimal (10, 11). Second, PFTs and routine hemostatic assays do not evaluate the fibrinolytic properties in a patient. Reduced susceptibility to fibrinolysis was recognized as an independent risk factor for recurrent ischemia in patients recovering from acute CAD (12, 13). Hence, a whole blood assay to quickly identify patients with diminished fibrinolytic potential might be of interest for more accurate risk assessment in patients post-PCI.

No global assays of hemostasis are currently implemented in drug monitoring guidelines to evaluate the overall effect of antithrombotic treatment strategies and potential additional hemostatic or fibrinolytic abnormalities. Rotational thromboelastometry (ROTEM) is a whole blood point-of-care (POC) viscoelastic assay that provides a quick overview of global hemostatic parameters. It is currently recommended to guide blood transfusion in cardiovascular surgery, trauma care and post-partum hemorrhage (14). In addition to its applicability in transfusion management, new areas of interest are explored including characterizing septic coagulopathy and detecting the presence of antithrombotic drugs (15, 16). An additional tissue plasminogen activator (tPA) based ROTEM assay was recently developed to assess the dynamic properties of fibrinolysis after clot formation (17). These characteristics highlight the potential of ROTEM for hemostasis (and fibrinolysis) monitoring in post-PCI patients on antithrombotic drugs.

The primary aims of the current paper are 1) to evaluate (tPA) ROTEM results in vulnerable post-PCI patients on dual antithrombotic treatment, including the potential value of (tPA) ROTEM to identify the presence of anticoagulants and antiplatelet drugs, and 2) to evaluate the prognostic value of (tPA) ROTEM regarding the identification of post-PCI patients on DAPT at risk for bleeding or recurrent ischemic events.

## Methods

### Study Population

#### Antiplatelet Therapy Outpatient Clinic

The primary cohort study design has been described more extensively elsewhere (18). High clinical-risk patients using dual antithrombotic medication after elective or emergency PCI between April 2014 and January 2019 at Maastricht University Medical Centre+ (MUMC+) were included in this study. Vulnerable patients were referred to a specialized outpatient clinic within the Thrombosis Expertise Centre by one dedicated interventional cardiologist for assessment of on-treatment bleeding and ischemic risks. High clinical-risk patients were defined as patients who had ≥3 risk factors for bleeding or ischemic events, which include: old age (≥75 years), female gender, renal dysfunction (estimated Glomerular Filtration Ratio (eGFR) <60 mL/min), anemia at the time of PCI (<13.2 g/dL for men and <11.8 g/dL for women), low body weight (<60 kg), hypertension, diabetes mellitus, previous stroke, previous major bleeding, liver dysfunction, history of gastric/duodenal ulcers, previous in-stent thrombosis, high-risk stenting (multivessel PCI or main coronary artery thrombosis), daily use of non-steroidal anti-inflammatory drugs (NSAIDs) and/or use of serotonin reuptake inhibitors (SSRIs). Exclusion criteria for the general cohort were known platelet function disorders, coronary intervention or new ischemic event within 7 days before inclusion, signs of active infection during the visit to the outpatient department, non-compliance and withdrawal of informed consent. Additional exclusion criteria for the current analyses were no dual antithrombotic treatment at the time of ROTEM measurement and no ROTEM performed.

Dual antithrombotic treatment consisted of a P2Y12 inhibitor (clopidogrel, prasugrel, ticagrelor) in combination with either aspirin (DAPT) or an anticoagulant (VKA or DOAC). At the first outpatient visit approximately 1 month post PCI (T1) the clinician recollected medical history, bleeding/ischemic events and performed a medication check. Blood was drawn 3–8 h after last drug intake for laboratory assessment, including ROTEM. Patients were stratified in different antithrombotic treatment groups based on the treatment regimen at T1. At a second outpatient visit 6 months post PCI (T2) the occurrence of ischemic and/or bleeding events was re-evaluated. This study was approved by the medical ethical committee of the MUMC+ (NL38767.068.11, METC number 11-2-096) and written informed consent was obtained from all patients.

#### Control Group

The control group consists of a random sample of preoperative patients (*n* = 95) not using antithrombotic medication. The control group consisted of a subgroup from the study by Vries et al. defined as “patients not reporting bleeding symptoms” (19). Here, preoperative patients admitted at the MUMC+ for any elective surgery were included between September 2013 and January 2016. Exclusion criteria were age ≤ 18, known bleeding disorders, antithrombotic drug use, NSAID use, platelet count <100.000/mL, anemia, pregnancy or a positive anesthesiology bleeding questionnaire. Blood was withdrawn during the post-operative study visit where (tPA) ROTEM, complete blood count and renal function were determined among other things.

### Laboratory

Blood was collected in a 3.2% sodium-citrate tube and all laboratory tests were performed within 4 h after blood collection. Platelet poor plasma (PPP) was obtained by centrifugation at 2,500 g for 5 min, followed by centrifugation at 10,000 g for 10 min at 18°C.

#### Rotational Thromboelastometry

ROTEM is a whole blood assay that measures changes in viscoelastic properties during clot formation. In the current study, EXTEM, INTEM, FIBTEM and tPA ROTEM assays were performed on a ROTEM delta (Werfen; Barcelona, Spain). EXTEM and FIBTEM clot formation is triggered by tissue factor (extrinsic coagulation pathway), with FIBTEM containing additional cytocholasin D to eliminate platelet function. The INTEM assay is activated by kaolin and illustrates the intrinsic coagulation pathway. The following standard ROTEM parameters were analyzed: CT (clotting time in seconds), A5 (amplitude at 5 min in mm), A10 (amplitude at 10 min in mm), CFT (clot formation time in seconds), MCF (maximum clot firmness in mm), alpha angle (the angle between the middle axis and the tangent to the clotting curve through the 2 mm amplitude point), Ly30 (percentage lysis of MCF at 30 min in %), Ly45 (percentage lysis of MCF at 45 min in %) and Ly60 (percentage lysis of MCF at 60 min in %).

TPA ROTEM is an EXTEM-like assay where recombinant tissue plasminogen activator (r-tPA) is added to evaluate the fibrinolytic properties of a clot. The assay was previously validated by Kuiper et al. (20). In short, 125 ng/mL tPA was added in addition to 35 pM TF to induce clot formation and breakdown simultaneously. Additional ROTEM parameters specific to the tPA assay include LOT (lysis onset time; time from CT until a 15% drop in MCF) and LT (lysis time; time from CT until a 90% drop in MCF). If LOT and/or LT were not reached within 2 h due to limited clot breakdown, results were capped at 7,200 seconds.

#### Other Laboratory Parameters

Fibrinogen level (Clauss method, Thrombin reagent, Siemens), PT (Innovin Pt, Siemens) and activated partial thromboplastin time (aPTT; Actine FSL, Siemens) were determined in PPP on a Sysmex CS2100i.

### Clinical Outcomes

We aimed to explore whether ROTEM parameters at T1 could identify patients on DAPT at risk for developing clinically relevant bleeding or major adverse cardiovascular events (MACE) until the 6-month follow-up (T2) and compared them with routine hemostasis assays (PT, aPTT, fibrinogen and platelet count). The VKA+P2Y12i and DOAC+P2Y12i groups were not analyzed for clinical outcomes due to the limited number of patients available. Bleeding events were recorded using the Bleeding Academic Research Consortium (BARC) criteria, which contains unified and validated bleeding criteria (21, 22). Clinically relevant bleeding was defined as BARC type ≥2. In addition, recurrent ischemic events were recorded during the follow-up visits and MACE was defined as myocardial infarction, stroke, and all-cause mortality. Both bleeding and ischemic endpoints were assessed at 1 month (T1) and 6 months (T2) after PCI. The analysis was performed in patients treated with DAPT for ≥6 months. Exclusion criteria were clinically relevant bleeding (BARC type ≥2) or MACE prior to ROTEM measurement at T1, switch to an oral anticoagulant (OAC) before T2 and no T2 follow-up.

### Statistical Analysis

The statistical analyses were performed in IBM SPSS Statistics 25.0 for Windows and figures were plotted in GraphPad Prism for Windows unless stated otherwise. Normality was determined by visual assessment and the Shapiro-Wilk test. Normally distributed continuous variables are presented as mean with standard deviation (SD) and non-parametric data are presented as median with interquartile range (IQR). At baseline, statistical significance was determined between the control vs post-PCI group and within the different medication groups using Mann-Whitney U, Kruskal-Wallis and chi-squared test as appropriate. The Kruskal-Wallis test was applied to evaluate differences in ROTEM parameters between antithrombotic treatment groups. When significant (*p* < 0.05), follow-up Mann-Whitney U tests were used for pairwise comparisons. To adjust for multiple testing, a Bonferroni correction was applied and effects are reported at a (0.05/6=) 0.008 level of significance.

The discriminating ability of ROTEM parameters and routine hemostasis assays (PT and aPTT) to identify patients on anticoagulant treatment (VKA + DOAC, VKA separately, and DOAC separately) was assessed using receiver operating characteristics (ROC). Both the control group and patients on DAPT were included as patients not receiving anticoagulant treatment. The Youden index was calculated (J = sensitivity + specificity −1) to determine the cut-off value for maximized sensitivity and specificity. The area under the curve (AUC) was compared using DeLong test in R. In an attempt to evaluate the prognostic value of ROTEM, the Mann-Whitney U test was used for comparison of T1 ROTEM parameters between patients with and without clinically relevant bleeding or MACE until T2. In cases where significant differences were achieved (*p* < 0.05), discriminative performance was further assessed by ROC analysis.

## Results

### Study Population

**Figure 1:**
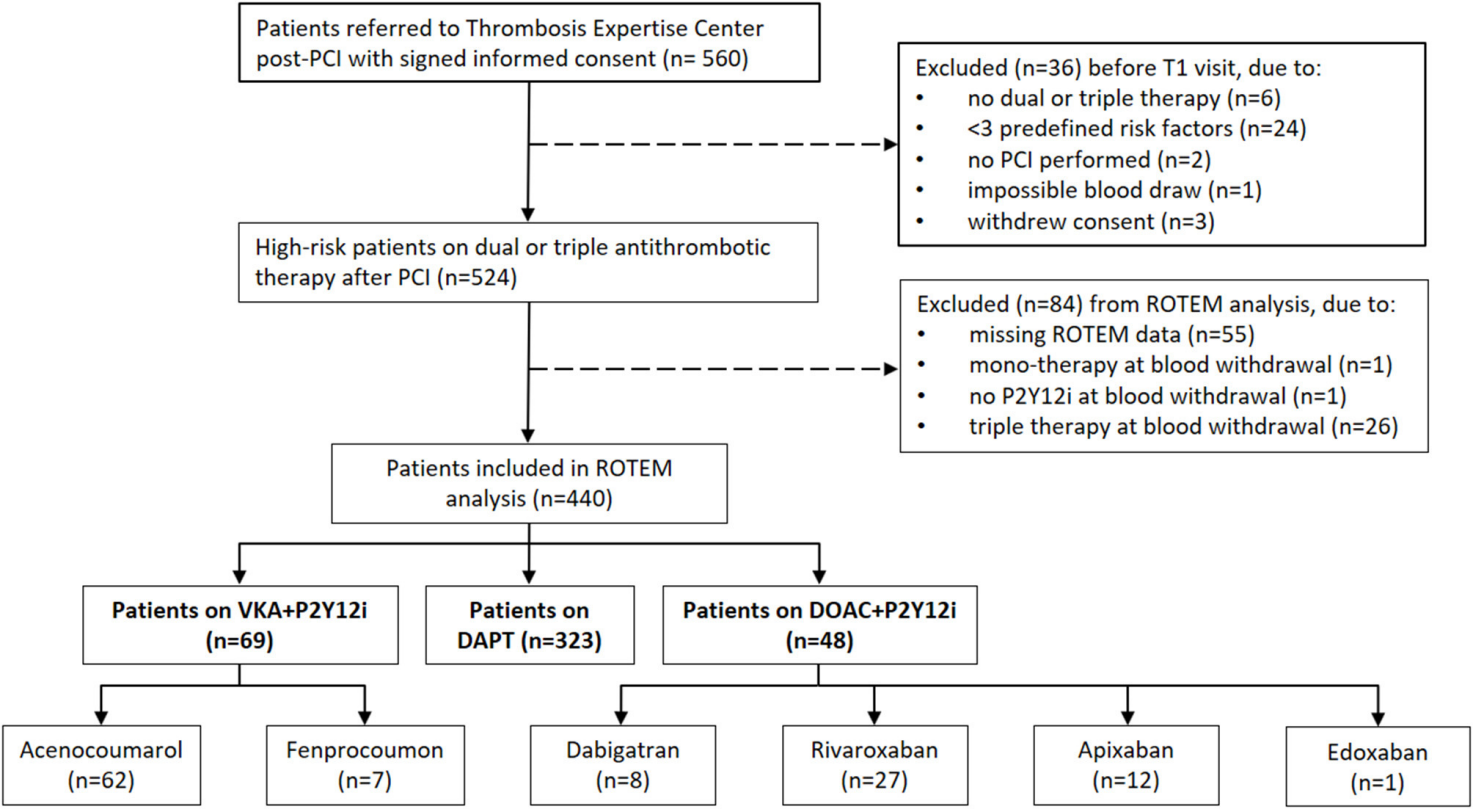
Flow diagram of study inclusion and stratification in treatment groups. DAPT, dual antiplatelet therapy consisting of aspirin and a P2Y12-inhibitor (P2Y12i); DOAC, direct oral anticoagulant; PCI, percutaneous coronary intervention; ROTEM, rotational thromboelastometry; VKA, vitamin K antagonist.

The initial cohort consisted of 560 high-risk patients undergoing PCI at the MUMC+ between May 2014 and May 2019 (18). As illustrated in Figure 1, 440 patients were suitable for analysis. Demographics, baseline characteristics and routine laboratory values of the antithrombotic treatment and control groups are presented in Table 1. In general, the control group appears younger and with fewer comorbidities (e.g. diabetes) compared to the post-PCI population. Patients on anticoagulants often had a medical history with atrial fibrillation and both PT and aPTT were generally prolonged in patients receiving a DOAC or VKA. Most patients received DAPT (*n* = 323, 73.4%), followed by VKA in combination with a P2Y12i (VKA+P2Y12i; *n* = 69, 15.7%) and DOAC in combination with a P2Y12i (DOAC+P2Y12i; *n* = 48, 11.1%). In the VKA+P2Y12i and DOAC+P2Y12i group 12 (17.4%) and 14 (28.6%) patients started with triple antithrombotic therapy, respectively, which was converted to dual antithrombotic treatment prior to the first outpatient clinic visit (T1). Patients were seen at T1 after a median [IQR] of 45 [36–56] days post-PCI.

**Table 1:** Demographics, baseline characteristics and routine laboratory values of the control, DAPT, VKA+P2Y12i and DOAC+P2Y12i groups.

**Mean (st. dev.)Median [IQR]**	**Control**	**DAPT**	**VKA + P2Y12i**	**DOAC + P2Y12i**	***p*-value: control vs**	***p*-value:**
***n* (%)**	***n =* 95**	***n =* 323**	***n =* 69**	***n =* 48**	**post-PCI patients**	**medication groups**
***Patient characteristics***						
Age (years)	57 [47–67]	76 [69–81]	78 [72–80]	75 [71–80.75]	0.000	0.552
Gender (man)	53 (55.8)	165 (51.1)	50 (72.5)	32 (66.7)	0.951	0.002
BMIΦ	26.0 [24.1–28.4]	26.6 [24.1–30.0]	27.6 [24.7–30.3]	26.9 [23.8–30.0]	0.065	0.758
Smoking (yes)Φ	22 (23.2)	45 (14.0)	7 (10.3)	7 (14.6)	0.018	0.697
Diabetes (yes)	5 (5.3)	120 (37.2)	23 (33.3)	15 (31.3)	0.000	0.648
Hypertension (yes)	ND	274 (84.8)	58 (84.1)	41 (85.4)		0.978
Previous stroke (CVA and/or TIA)Φ	ND	80 (24.8)	22 (31.9)	15 (31.3)		0.364
Previous PCI	ND	121 (37.5)	26 (37.7)	20 (41.7)		0.854
Atrial fibrillation in medical history	ND	7 (2.2)	52 (75.4)	45 (93.8)		0.000
***Laboratory values***						
Platelets (10^∧^9/L)	259 [226–299]	252 [214–296]	245 [195–303]	248.0 [212.75–311.5]	0.286	0.542
Leukocytes (10^∧^9/L)	6.9 [5.6–8.1]	7.6 [6.4–9.3]	7.3 [6.7–8.6]	7.50 [6.3–8.4]	0.000	0.595
Hematocrit (L/L)	0.43 [0.41–0.45]	0.40 [0.37–0.43]	0.41 [0.37–0.44]	0.41 [0.38–0.44]	0.000	0.426
Hemoglobin (mmol/L)	9.0 (0.8)	8.2 (1.0)	8.3 (1.1)	8.2 (1.1)	0.000	0.737
PT (s)Φ	10.3 [10.1–10.5]	10.6 [10.3–11.0]	25.5 [21.4–32.0]	12.7 [11.4–14.4]	0.000	0.000
aPTT (s)Φ	26 (25–27)	26.0 (25–27)	36 (34–38)	33.0 [29–36.75]	0.000	0.000
Fibrinogen (g/L)Φ	3.2 [2.8–3.6]	3.7 [3.1–4.2]	3.8 [3.3–4.2]	3.5 [3.0–4.0]	0.000	0.453
***P2Y12 inhibitor***						
Clopidogrel		218 (67.5)	68 (98.6)	43 (89.6)		0.000
Prasugrel		53 (16.4)	1 (1.4)	1 (2.1)		
Ticagrelor		52 (16.1)	0 (0)	4 (8.3)		
***Other antithrombotic medication***						
Acenocoumarol			62 (89.9)			
Fenprocoumon			7 (10.1)			
Rivaroxaban				27 (56.3)		
Dabigatran				8 (16.7)		
Apixaban				12 (25.0)		
Edoxaban				1 (2.1)		
***Index PCI***						
Acute		219 (67.8)	34 (49.3)	31 (64.6)		0.014
Elective		104 (32.2)	35 (50.7)	17 (35.4)		
Days between ROTEM and PCI		46 (36–56)	47 (36–60)	44 [37.25–53.75]		0.824

*Patient characteristics and laboratory values as presented at the outpatient department of vascular medicine one month post-PCI visit (T1; DAPT, VKA + P2Y12i, DOAC + P2Y12i groups) and the post-operative study visit (control group). PCI, percutaneous coronary intervention; BMI, body mass index; SD, standard deviation; IQR, interquartile range; PT, prothrombin time; aPTT, activated partial thromboplastin time; ND, not determined. Φ Data are missing for BMI (DAPT n = 6), smoking (DAPT n = 2, VKA+P2Y12i n = 1), previous stroke (CVA and/or TIA) (DAPT n = 1), PT/aPTT/Fibrinogen (control n = 1, DAPT n = 2)*.

### Rotational Thromboelastometry

**Figure 2:**
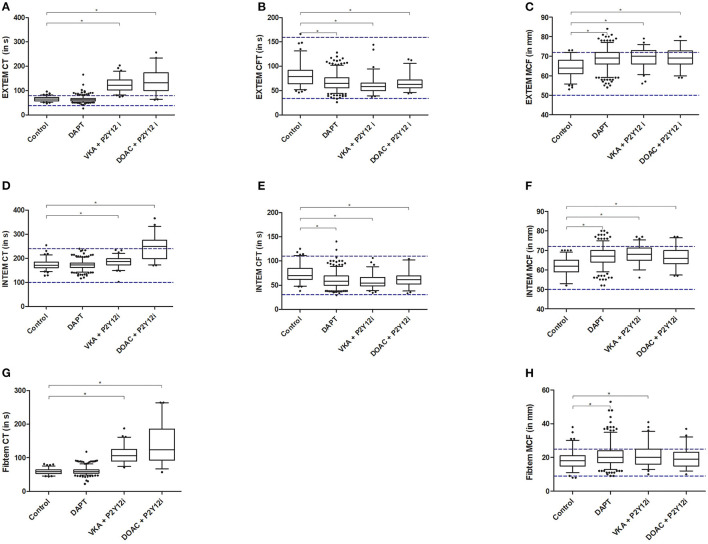
EXTEM **(A–C)**, INTEM **(D–F)** and FIBTEM **(G,H)** results for clotting time (CT; **A,D,G**), clot formation time (CFT; **B,E**) and maximum clot firmness (MCF; **C,F,H**). Presented are median, IQR and 5–95 percentile whiskers. Dashed lines illustrate reference ranges according to the manufacturer. Significant differences (*p* < 0.008) compared to the control group are reported with an asterisk.

**Figure 3:**
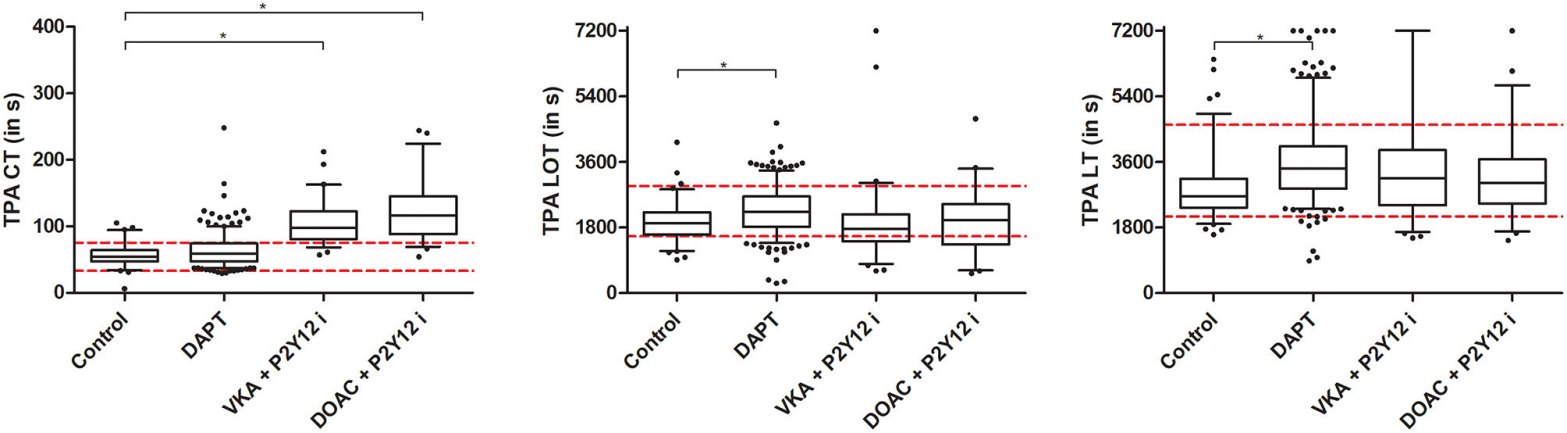
Clotting time (CT), lysis onset time (LOT) and lysis time (LT) tissue plasminogen activator (tPA) ROTEM. Presented are median, IQR and 5–95 percentile whiskers. Dashed lines illustrate tPA ROTEM reference range as determined by Kuiper et al. (20) Significant differences (*p* < 0.008) compared to the control group are reported with an asterisk.

All ROTEM assays showed significantly prolonged CT in patients receiving anticoagulants (VKA+P2Y12i and DOAC+P2Y12i) when compared to the control group and patients receiving DAPT (Figures 2A,D,G, Supplementary Table 1). Almost all patients on anticoagulants exceeded the manufacturer's reference range for EXTEM CT. In general, CFT was shorter and A5, A10, and MCF were increased in post-PCI patients compared to the control group, irrespective of antithrombotic treatment in both the EXTEM and INTEM ROTEM assays (Figures 2C,F). The effect was less pronounced for the FIBTEM assay, as clot stiffness parameters were not consistently increased in all post-PCI antithrombotic treatment groups when compared to control (Supplementary Table 1). Furthermore, fibrinogen was elevated and PT was prolonged in post-PCI patients, though the DAPT group showed limited PT prolongation compared to control (10.6 [10.3–11.0] vs. 10.3 [10.1–10.5]; *p* < 0.001, Supplementary Figure 1A). Fibrinolysis parameters (LOT and LT) were significantly prolonged in DAPT patients compared to the control population. However, VKA and DOAC +P2Y12i did not show a similar profile and the previously determined reference ranges for LOT and LT were generally not exceeded in any group (Figures 3B,C).

ROC-analysis revealed that EXTEM and FIBTEM CT had good discriminating capacity to detect the presence of anticoagulants with an AUC of 0.979 and 0.978, respectively (Table 2). However, both assays did not perform significantly better than the routine PT. INTEM and tPA CT had poorer discriminating ability compared to PT, evidenced by the significantly lower AUC (*p* < 0.001). When analyzed separately, PT outperformed all ROTEM CT assays in the detection of patients on VKA+P2Y12i treatment (Supplementary Table 2). Furthermore, the discriminating capacity to detect the presence of DOACs was similar for PT and EXTEM CT with an AUC of 0.929 and 0.963, respectively (Supplementary Table 3). However, EXTEM CT in the DOAC+P2Y12i group prolonged relatively more compared to control (Figure 2A) than the PT (Supplementary Figure 1A).

**Table 2:** ROC analysis for anticoagulant presence.

	**AUC**	**Youden's index**	**Sensitivity**	**Specificity**	***p*-value**
PT^*^	0.971	11.35	92.3%	90.8%	Reference
aPTT^*^	0.965	28.5	92.3%	89.2%	0.48
**EXTEM CT**	0.979	80.5	94.9%	94.5%	0.39
**INTEM CT**	0.791	180.5	71.8%	73.2%	<0.001
**FIBTEM CT**	0.978	74.5	90.6%	92.1%	0.47
**tPA CT** **Φ**	0.916	78.5	82.1%	83.2%	<0.001

* *PT and aPTT missing for 3 patients who did not receive anticoagulant treatment. Φ tPA CT missing for 1 patient who did not receive anticoagulant treatment*.

In summary, ROTEM CT was significantly prolonged in patients using anticoagulants and, though not outperforming PT, EXTEM CT had good discriminating ability to detect the presence of both VKAs and DOACs. Additionally, ROTEM clot firmness was generally increased in all post-PCI patients and ROTEM LOT and LT demonstrated diminished fibrinolysis in the DAPT group only.

### Clinical Outcomes in Patients Using DAPT

The current analysis was performed on patients using DAPT only (*n* = 323). Patients were excluded from the current analysis due to clinically relevant bleeding or MACE prior to ROTEM measurement at T1 (*n* = 28), switch to an oral anticoagulant before T2 (*n* = 3), DAPT <6 months (*n* = 32), and no T2 follow-up (*n* = 33) (several patients presented with >1 exclusion criteria). Thus, 252 patients (78%) were eligible for follow-up analysis of clinically relevant bleeding or MACE. The second outpatient visit (T2) took place 154 [146–168] days after T1. Thirteen patients (5.2%) developed MACE during T1-T2 follow-up (non-STEMI; *n* = 4, CVA/TIA; *n* = 2, all-cause death; *n* = 7). Neither ROTEM parameters nor routine hemostasis assays at T1 significantly differed between patients with and without MACE. Thirteen patients (5.2%) developed clinically relevant bleeding between T1 and T2 (BARC 2; *n* = 10, BARC 3; *n* = 3). Statistical significance between patients with and without clinically relevant bleeding was achieved for EXTEM CT (68 [62–70] vs. 62 [57–68], *p* = 0.030). Routine hemostasis assays (PT, aPTT, fibrinogen and platelet count) were unable to discern patients at risk for clinically relevant bleeding. ROC analysis revealed a moderate discriminative ability of EXTEM CT to detect clinically relevant bleeding during T1-T2 follow-up, with an AUC of 0.679 (*p* = 0.030). ROTEM values and routine hemostasis assays stratified by bleeding and MACE are presented in Supplementary Tables 4 and 5, respectively.

## Discussion

The current study evaluated routine and tPA ROTEM results in a vulnerable post-PCI population on dual antithrombotic treatment and yielded four main findings: (1) EXTEM CT had good discriminating capacity for the detection of anticoagulants, though not outperforming PT. (2) All post-PCI patients, irrespective of antithrombotic treatment, had increased clot formation as evidenced by shortened CFT and increased MCF one month after intervention. (3) Fibrinolysis was diminished in patients on DAPT treatment, but not in the anticoagulant treatment groups. (4) EXTEM CT could discriminate between patients with and without clinically relevant bleeding (BARC ≥ 2) over a five-month follow-up in the DAPT treatment group.

We assessed whether ROTEM could identify patients using antithrombotic treatment. Currently, there is an unmet clinical need for a global screening assay, such as ROTEM, to identify the presence of antithrombotic medication in emergency situations, such as severe trauma, massive hemorrhage, stroke, and urgent surgery. An unknown unconscious or delusional patient, unavailable for anamnestic evaluation, could present with severe (i.e. intracranial) bleeding or could be awaiting thrombolytic therapy in stroke whilst under antithrombotic treatment. It is essential to screen for the presence of these drugs to provide appropriate hemostatic interventions when required. The ideal global screening assay should be able to detect clinically relevant levels of antithrombotic drugs, have a short turn-around time, and be available on-demand. Our results showed clear ROTEM CT prolongation in the presence of anticoagulants, specifically in the EXTEM, FIBTEM and tPA assays. Contrarily, ROTEM CT was similar between the control group and patients receiving antiplatelet medication only in the DAPT group. In line with our observation that ROTEM is unable to identify antiplatelet drugs, the general insensitivity of ROTEM to detect platelet function was previously reported (17). Another study in patients receiving DAPT (*n* = 78) did show a significant EXTEM and INTEM CT prolongation when compared to healthy controls (39). However, there was evident overlap between the DAPT and control group in CT values and, thus, ROTEM would still be considered unsuitable to identify patients using antiplatelet drugs. Similarly, no differences in ROTEM parameters was observed between patients using 75mg vs 150 mg clopidogrel one month after PCI (40). A novel ROTEM assay, ROTEM platelet impedance aggregometry (ROTEM-PLT), has recently become available to evaluate antiplatelet therapy in patients (34). However, this novel ROTEM platelet assay was not evaluated in the current study. As previously acknowledged, the effect of anticoagulants was clearly evidenced by the CT prolongation in all ROTEM assays and subsequent ROC analysis revealed that EXTEM and FIBTEM CT had excellent discriminative ability between patients that do (not) receive anticoagulants. *In vitro* spiking studies demonstrated a DOAC dose-response effect in ROTEM CT, which was most pronounced for the EXTEM assay (35). Similar effects were observed *in vivo* in patients treated with a DOAC or VKA (36, 39, 41–43), hinting that ROTEM may be suitable to monitor anticoagulant drug concentrations. Although EXTEM CT had almost perfect discriminatory ability in the current setting, the observed AUC (0.979) did not outperform the PT assay (Table 2). However, ROTEM's whole blood, on-demand availability and other POC characteristics make it more suitable in emergency situations. Therefore, ROTEM analysis might be convenient in an unknown, unconscious patient to quickly determine the presence of anticoagulants. Specifically for the detection of DOACs in acute situations, as no POC-assay is currently available for this purpose.

Post-PCI patients generally presented with increased clot stiffness one month after the intervention irrespective of antithrombotic treatment, illustrated by the shorter CFT and higher MCF values compared to control. This suggests a more universal pathology underlying increased clot stiffness in patients requiring PCI rather than an effect of antithrombotic treatment. For correct interpretation of these results, it is essential to realize that ROTEM clot stiffness (partly) depends on fibrinogen concentration. The fibrinogen concentration was elevated in all post-PCI treatment groups (Supplementary Figure 1), thus possibly explaining the rise in MCF. However, the lack of consistent FIBTEM clot stiffness increase, which is most dependent on fibrinogen due to cytochalasin D platelet inhibition, may point to an alternative mechanism. In recent years the association between clot architecture pathophysiology and clinical phenotypes has been reviewed in literature. It was established that patients with (recurrent) thrombosis, such as CAD and stroke, demonstrate abnormal clot architecture (44–46). Specifically, patients with thrombotic complications form dense fibrin networks with increased stiffness and limited permeability. It has been suggested that ROTEM could detect such subtle differences in clot architecture. Two studies that evaluated ROTEM clot stiffness in plasma with different fibrinogen entities and a fibrinogen cross-linking polymer support that ROTEM is susceptible to changes in clot architecture (37, 38). The rise in clot stiffness in post-PCI patients compared to controls may therefore originate from both the elevated fibrinogen concentration and the abnormal clot architecture associated with CAD.

The described clot abnormalities in patients with thrombosis, higher density and reduced permeability, predispose to limited clot breakdown as the formed fibrin fibers are less accessible for tPA-mediated fibrinolysis (23, 24). Additionally, patients with thrombosis show increased levels of plasminogen activator inhibitor-1 (PAI-1) (25, 44). Over the last two decades strong connections between abnormal fibrin structures, fibrinolysis and thrombotic complications were identified (23, 26, 44–46). Farag et al. demonstrated that POC measurement of fibrinolysis (Global Thrombosis Test) could aid in identifying STEMI patients undergoing PCI at risk for recurrent thrombosis (12). Similarly, Sumaya et al. evaluated fibrinolysis in acute coronary syndrome patients with a turbidimetric assay and found that diminished fibrinolysis was associated with myocardial infarction, cardiovascular death, and all-cause death (13). Our tPA ROTEM did not illustrate a similar predictive ability for MACE and no significant difference in fibrinolysis parameters between the control, VKA+P2Y12i and DOAC+P2Y12i groups was observed. However, the DAPT patient group had significantly prolonged LOT and LT reflecting a decreased fibrinolytic potential. Of note, patients included in the studies of Farag and Sumaya et al. received no anticoagulants at blood withdrawal. Therefore, the lack of LOT and LT prolongation in our anticoagulant treatment groups may arise from the effect of DOACs and VKA on fibrinolysis. Indeed, DOACs enhance fibrinolysis by (indirectly) inhibiting thrombin and, consequently, thrombin-activatable fibrinolysis inhibitor (TAFI) (27–30). Additionally, the FXa inhibitors apixaban and rivaroxaban enable the tPA cofactor function of a pro-fibrinolytic FXa breakdown product (31). Finally, all anticoagulants limit thrombin activity, increase clot permeability and, thereby, make fibrin fibers more available for degradation (32, 33). Taken together, the similar tPA ROTEM LOT and LT values in the anticoagulant therapy groups (DOAC+P2Y12i and VKA+P2Y12i) as in the control patients might be explained by the described anticoagulant-mediated pro-fibrinolytic mechanism that overrules any potential natural hypofibrinolytic phenotype in these patients. A limitation to our study is that no sample prior to the treatment start was obtained to distinguish between the native hemostasis profile and the effect of the antithrombotic drugs on an individual level.

To the best of our knowledge, there are no studies available that evaluated the long-term potential of ROTEM to predict bleeding. We found a predictive ability of EXTEM CT for the development of clinically relevant bleeding over five months in post-PCI patients on DAPT treatment. EXTEM CT was significantly prolonged in patients with clinically relevant bleeding when compared to non-bleeders. Furthermore, ROC analysis illustrated a moderate discriminative ability with an AUC of 0.679. FIBTEM and tPA CT showed a similar trend, suggesting that use of an extrinsic activator (tissue factor) reveals the bleeding phenotype in these patients. However, since routine hemostasis assays, including tissue factor activated PT, did not differ between bleeders and non-bleeders, this aspect seems unique to ROTEM assays. This might introduce a novel application of viscoelastic testing to better identify patients at risk for bleeding in addition to the “classic” risk factors. However, results should be interpreted with care as only 13 patients developed clinically relevant bleeding. Additionally, VKA+P2Y12i and DOAC+P2Y12i medication groups were not evaluated due to the limited number of patients. Larger studies evaluating the ability of ROTEM to predict long-term bleeding complications in patients on antithrombotic medication are therefore required.

Our study has several limitations. First, a control group of similar age and comorbidities without any antithrombotic medication was unavailable for analysis. Second, though patient compliance was checked verbally and with the pharmacy, correct intake of antithrombotic drugs prior to the blood withdrawal cannot be confirmed. Third, patients received their antithrombotic treatment strategy based on clinical risk factors, e.g. patients with atrial fibrillation receive an oral anticoagulant, introducing classification bias inherent to the real-world setting. Fourth, patients who developed bleeding complications and/or MACE in the first month prior to T1 could not be included in the follow-up analysis. Since most events occurred during the first month post-PCI earlier ROTEM assessment may yield valuable results in future studies. Furthermore, a longer follow-up (≥1 year) and inclusion of lower clinical risk patients would be of interest in future studies. Nonetheless, this is the first ROTEM study performed in a large post-PCI high-risk cohort with long-term follow-up of ischemic and bleeding complications.

In conclusion, ROTEM has high potential for identifying the presence of anticoagulants in acute situations. Furthermore, tPA ROTEM did illustrate diminished fibrinolysis in CAD patients receiving DAPT, but no association with clinical outcomes was observed. We did observe a moderate predictive ability of EXTEM CT to identify patients at risk for clinically relevant bleeding, which may be of interest to help guide bleeding risk assessment in patients post-PCI. These findings could serve as a stepping stone for further exploration of ROTEM in emergency situations and long-term risk assessment.

## Data Availability Statement

The datasets presented in this article are not readily available because no concrete agreements on data sharing have been made yet. Before any data is shared outside the MUMC+, a datasharing plan will be drawn up, in consultation with the data officer, that conforms to relevant laws and regulations concerning personal data. Requests to access the datasets should be directed to Renske H. Olie, renske.olie@mumc.nl.

## Ethics Statement

The studies involving human participants were reviewed and approved by NL38767.068.11, METC number 11-2-096. The patients/participants provided their written informed consent to participate in this study.

## Author Contributions

AH contributed to the design of the manuscript, performed statistical analysis, and was main author. RO, YH, MV, and HC developed the design of the study cohort. RO and MV collected clinical data and PV performed laboratory analysis. RO, MV, PM, HC, and YH critically reviewed the manuscript and revised the intellectual content. All authors contributed to the article and approved the submitted version.

## Funding

This work was supported by the Thrombosis Expertise Centre at the Heart and Vascular Centre of Maastricht University Medical Centre (MUMC+) in the Netherlands. This research received no specific grant from any funding agency or commercial sector.

## Conflict of Interest

RO has received research support an honoraria from Bayer, Pfizer/BMS, Leo Pharma, Portola, and Sanofi. HC has received grants from Bayer and Pfizen, is a consultant for Alveron, and a shareholder of Coagulation Profile. YH has received ROTEM cartridges free of charge for previous research not related to the current manuscript. The remaining authors declare that the research was conducted in the absence of any commercial or financial relationships that could be construed as a potential conflict of interest.

## Publisher's Note

All claims expressed in this article are solely those of the authors and do not necessarily represent those of their affiliated organizations, or those of the publisher, the editors and the reviewers. Any product that may be evaluated in this article, or claim that may be made by its manufacturer, is not guaranteed or endorsed by the publisher.
