# Supplementary material for: Rotational Thromboelastometry in High-Risk Patients on Dual Antithrombotic Therapy After Percutaneous Coronary Intervention
Source: Front Cardiovasc Med. 2021 Dec 22;8:788137. doi: 10.3389/fcvm.2021.788137 (PMC8727359; doi:10.3389/fcvm.2021.788137)
Supplement: Supplementary file 1 [file Data_Sheet_1.docx]

Supplementary Material

# Supplementary Figures

**Supplementary Figure 1.** Prothrombin time (PT; A), activated partial thromboplastin time (aPTT; B), fibrinogen (C) and platelet count (D). Presented are median, IQR and 5-95 percentile whiskers. Significant differences (p<0.008) compared to the control group are reported with an asterisk.

# Supplementary Tables

**Supplementary Table 1.** EXTEM, INTEM, FIBTEM and tPA ROTEM parameters in the control, DAPT, P2Y12i+VKA and P2Y12i+DOAC groups

| Median [IQR] | **Control group (n=95)*** | **DAPT (n=323)**** | **P2Y12i + VKA (n=69)ᶲ** | **P2Y12i + DOAC (n=48)ᶲᶲ** |
| --- | --- | --- | --- | --- |
| ***EXTEM*** | | | | |
| **CT** | 65 [58-71] | 63 [57-68] | 122 [103-144] ^a,b^ | 132 [100-173] ^a,b^ |
| **A5** | 47 [43-52] | 53 [49-58] ^a^ | 56 [52-60] ^a,b^ | 55 [51-59] ^a^ |
| **A10** | 57 [54-61] | 62 [59-67] ^a^ | 65 [60-68] ^a^ | 64 [60.25-67.75] ^a^ |
| **CFT** | 79 [64-92] | 65 [56-76] ^a^ | 58 [50-65] ^a,b^ | 63 [56-71.75] ^a^ |
| **MCF** | 64 [61-68] | 69 [66-72] ^a^ | 70 [66-73] ^a^ | 69 [66-72.25] ^a^ |
| **Alpha angle** | 75 [71-77] | 77 [75-79] ^a^ | 78 [77-80] ^a,b^ | 77.5 [76-79] ^a^ |
| **Li30** | 100 [99-100] | 100 [100-100] ^a^ | 100 [100-100] ^a^ | 100 [100-100] |
| **Li45** | 96 [94-98] | 98 [96-99] ^a^ | 98 [97-99] ^a^ | 97 [95.25-99] |
| **Li60** | 92 [90-94] | 94 [92-96] ^a^ | 94 [92-96] ^a^ | 93.5 [92-96] ^a^ |
| ***INTEM*** | | | | |
| **CT** | 170 [161-184] | 172 [162-181] | 187 [173-197] ^a,b^ | 249 [197.75-275.75] ^a,b,c^ |
| **A5** | 47 [43-51] | 52 [48-56] ^a^ | 54 [50-59] ^a^ | 53 [48-55.75] ^a^ |
| **A10** | 57 [53-60] | 61 [58-65] ^a^ | 63 [59-67] ^a^ | 61.5 [57-64.75] ^a^ |
| **CFT** | 70 [62-84.25] | 58 [50-69] ^a^ | 54 [49-66] ^a^ | 62 [52-69.5] ^a^ |
| **MCF** | 62 [59-65] | 67 [64-70] ^a^ | 68 [65-71] ^a^ | 66.5 [63.25-70] ^a^ |
| **Alpha angle** | 76 [73-77] | 78 [76-80] ^a^ | 79 [76-80] ^a^ | 77.5 [75-79] ^a^ |
| **Li30** | 99 [98-100] | 100 [99-100] ^a^ | 100 [100-100] ^a^ | 100 [99-100] ^a, c^ |
| **Li45** | 94 [92-96] | 97 [95-98] ^a^ | 98 [96-98] ^a^ | 96 [94-98] ^a^ |
| **Li60** | 91 [88-93] | 94 [91-96] ^a^ | 94 [93-96] ^a^ | 93 [90-96] ^a^ |
| ***FIBTEM*** | | | | |
| **CT** | 57 [53-64] | 59 [54-64] | 106 [90-125] ^a,b^ | 124 [93-185.25] ^a,b^ |
| **A5** | 16 [13-19] | 18 [15-21] ^a^ | 17 [14-22] | 16 [12.25-19] ^b^ |
| **A10** | 17 [15-20] | 19 [16-23] ^a^ | 19 [15-23] | 17.5 [14-20.75] |
| **MCF** | 18 [15-21] | 20 [17-24] ^a^ | 20 [16-25] ^a^ | 19 [15-23] |
| **Li30** | 100 [99-100] | 100 [100-100] ^a^ | 100 [100-100] ^a^ | 100 [100-100] |
| **Li45** | 100 [97-100] | 100 [99-100] ^a^ | 100 [100-100] ^a^ | 100 [100-100] ^a^ |
| **Li60** | 100 [95-100] | 100 [98-100] ^a^ | 100 [100-100] ^a^ | 100 [99-100] ^a^ |
| ***TPA*** | | | | |
| **CT** | 54 [47-63] | 59 [47-74] | 97 [81-119] ^a,b^ | 116 [88-145.25] ^a,b^ |
| **A5** | 46 [39-51] | 53 [46-57] ^a^ | 53 [47-58] ^a^ | 52.5 [41.25-57] ^a^ |
| **A10** | 54 [47-59] | 60 [54-65] ^a^ | 60 [53-65] ^a^ | 58 [48-65] |
| **CFT** | 70 [58-95] | 59 [51-77] ^a^ | 63 [56-79] | 66.5 [58.25-102.5] ^b^ |
| **MCF** | 58 [50-62] | 64 [58-69] ^a^ | 61 [54-67] | 61 [48.5-67] |
| **Alpha angle** | 76 [72-78] | 78 [75-79] ^a^ | 77 [74-79] ^b^ | 76.5 [69.5-78.75] ^b^ |
| **Li30** | 90 [68-96] | 96 [86-99] ^a^ | 83 [52-95] ^b^ | 92 [53-97.75] ^b^ |
| **Li45** | 7 [1-50.25] | 60 [20-83] ^a^ | 29 [4-64] | 33 [4.25-75] |
| **Li60** | 1 [0-3.25] | 6 [2-35) ^a^ | 3 [1-18] | 2.5 [1-19] |
| **LOT** | 1918 [1606-2219] | 2227 [1823-2651]^a^ | 1755 [1429-2133]b | 2004 [1328-2435]^b^ |
| **LT** | 2649 [2345-3117] | 3414 [2867-4025]^a^ | 3133 [2405-3928] | 3019 [2459-3669]^b^ |

a. control vs treatment group p<0.008

b. DAPT vs anticoagulant + P2Y12i p<0.008

c. VKA + P2Y12i vs DOAC + P2Y12i p<0.008

*Values missing in the control group were INTEM CFT (n=1); tPA Li45, Li60 and LT (n=1)

**Values missing in the DAPT patient group were EXTEM Li60 (n=1), INTEM Li60 (n=1), tPA CT, A5, A10, CFT, MCF, Alpha angle, Li30, Li45 (n=1), Li60 (n=2), LOT (n=7), LT (n=11)

ᶲ Values missing in the VKA + P2Y12i patient group tPA Li60 and LT (n=1)

ᶲᶲ Values missing in the DOAC + P2Y12 patient group were FIBTEM MCF, Lit30, Li45 and Li60 (n=1); TPA LOT and LT (n=1)

**Supplementary Table 2.** ROC analysis for VKA presence

|  | **AUC** | **Youden’s index** | **Sensitivity** | **Specificity** | **p-value** |
| --- | --- | --- | --- | --- | --- |
| **PT*** | 1.000 | 14.45 | 1.000 | 1.000 | ref |
| **aPTT*** | 0.988 | 29.5 | 1.971 | 0.959 | 0.10 |
| **EXTEM CT** | 0.990 | 82.5 | 0.957 | 0.957 | 0.013 |
| **INTEM CT** | 0.697 | 178.5 | 0.652 | 0.682 | <0.001 |
| **FIBTEM CT** | 0.986 | 75.5 | 0.929 | 0.928 | <0.001 |
| **tPA CTᶧ** | 0.903 | 76.5 | 0.800 | 0.797 | <0.001 |

*PT and aPTT missing for 3 patients who did not receive VKA treatment

ᶧ tPA CT missing for 1 patient who did not receive VKA treatment

**Supplementary Table 3.** ROC analysis for DOAC presence

|  | **AUC** | **Youden’s index** | **Sensitivity** | **Specificity** | **p-value** |
| --- | --- | --- | --- | --- | --- |
| **PT*** | 0.929 | 11.15 | 0.854 | 0.843 | Ref |
| **aPTT*** | 0.933 | 28.5 | 0.833 | 0.892 | 0.76 |
| **EXTEM CT** | 0.963 | 80.5 | 0.938 | 0.945 | 0.13 |
| **INTEM CT** | 0.926 | 186.5 | 0.813 | 0.816 | 0.89 |
| **FIBTEM CT** | 0.967 | 69.5 | 0.938 | 0.873 | 0.095 |
| **tPA CTᶧ** | 0.933 | 84.5 | 0.833 | 0.890 | 0.89 |

*PT and aPTT missing for 3 patients who did not receive DOAC treatment

ᶧ tPA CT missing for 1 patient who did not receive DOAC treatment

**Supplementary Table 4:** ROTEM parameters of patients with and without clinically relevant bleeding (BARC≥2). P-values>0.1 were noted as not significant (Ns).

|  | **Patients without clinically relevant bleeding (n=239)** | **Patients with clinically relevant bleeding**  **(n=13)** | **P-value** |
| --- | --- | --- | --- |
| **EXTEM** | | | |
| CT (in s) | 62 [57-68] | 68 [62-70] | 0.030 |
| A5 (in mm) | 53 [49-57] | 55 [52-58] | Ns |
| A10 (in mm) | 62 [59-66] | 64 [61-66] | Ns |
| CFT (in s) | 65 [56-76] | 64 [54-68] | Ns |
| MCF (in mm) | 68 [66-72] | 69 [67-72] | Ns |
| Alpha angle (in °) | 77 [75-79] | 77 [76-79] | Ns |
| Li30 (in %) | 100 [100-100] | 100 [100-100] | Ns |
| Li45 (in %) | 98 [96-99] | 97 [96-98] | Ns |
| Li60 (in %) | 94 [92-96]* | 94 [91-96] | Ns |
| **INTEM** | | | |
| CT (in s) | 171 [162-182] | 177 [163-190] | Ns |
| A5 (in mm) | 52 [48-56] | 53 [50-56] | Ns |
| A10 (in mm) | 61 [58-65] | 62 [60-65] | Ns |
| CFT (in s) | 58 [50-69] | 60 [48-65] | Ns |
| MCF (in mm) | 67 [64-70] | 67 [66-69] | Ns |
| Alpha angle (in °) | 78 [76-80] | 78 [77-80] | Ns |
| Li30 (in %) | 100 [99-100] | 100 [99-100] | Ns |
| Li45 (in %) | 97 [95-98] | 97 [95-98] | Ns |
| Li60 (in %) | 94 [91-95]* | 93 [92-96] | Ns |
| **FIBTEM** | | | |
| CT (in s) | 59 [54-63] | 62 [57-71] | 0.058 |
| A5 (in mm) | 18 [15-21] | 19 [17-21] | Ns |
| A10 (in mm) | 19 [16-23] | 21 [19-23] | Ns |
| MCF (in mm) | 20 [17-24] | 22 [20-24] | Ns |
| Li30 (in %) | 100 [100-100] | 100 [100-100] | Ns |
| Li45 (in %) | 100 [100-100] | 100 [99-100] | Ns |
| Li60 (in %) | 100 [99-100] | 100 [97-100] | Ns |
| **TPA** | | | |
| CT (in s) | 57 [46-73]* | 74 [54-86] | 0.063 |
| A5 (in mm) | 52 [47-57]* | 50 [45-55] | Ns |
| A10 (in mm) | 60 [55-64]* | 58 [53-62] | Ns |
| CFT (in s) | 59 [51-76]* | 59 [55-82] | Ns |
| MCF (in mm) | 64 [59-69]* | 64 [55-66] | Ns |
| Alpha angle (in °) | 78 [75-79]* | 78 [73-79] | Ns |
| Li30 (in %) | 97 [86-99]* | 94 [92-98] | Ns |
| Li45 (in %) | 61 [20-83]* | 53 [19-71] | Ns |
| Li60 (in %) | 6 [2-30]** | 4 [2-15] | Ns |
| LOT (in s) | 2273 [1825-2651]ᶲ | 2080 [1966-2385] | Ns |
| LT (in s) | 3379 [2868-3977]ᶲᶲ | 3530 [3083-4074] | Ns |
| **Routine hemostasis assays** | | | |
| Fibrinogen (in g/L) | 3.6 [3.0-4.2]** | 3.4 [3.2-4.5] | Ns |
| PT (in s) | 10.6 [10.3-10.9]** | 10.7 [10.4-10.9] | Ns |
| aPTT (in s) | 26 [25-27]** | 26 [26-29] | Ns |
| Platelets (in 10^9^/L) | 248 [212-291] | 280 [244-287] | Ns |

*Missing value for 1 patient; ** missing values for 2 patients; ᶲ missing values for 7 patients; ᶲᶲ missing values for 10 patients

CT: Clotting Time, CFT: Clot Formation Time, AX: Amplitude at X minutes, MCF: Maximum Clot Firmness, LiX: Lysis index at X minutes, LOT: Lysis Onset Time, LT: Lysis Time, PT: prothrombin time, aPTT: activated partial thromboplastin time

**Supplementary Table 5:** ROTEM parameters of patients with and without major cardiovascular events (MACE). P-values>0.1 were noted as not significant (Ns).

|  | **Patients without MACE (n=239)** | **Patients with MACE (n=13)** | **P-value** |
| --- | --- | --- | --- |
| **EXTEM** | | | |
| CT (in s) | 63 [57-68] | 58 [55-61] | Ns |
| A5 (in mm) | 53 [49-58] | 53 [50-54] | Ns |
| A10 (in mm) | 62 [59-67] | 62 [59-63] | Ns |
| CFT (in s) | 65 [56-76] | 68 [63-76] | Ns |
| MCF (in mm) | 68 [66-72] | 68 [66-70] | Ns |
| Alpha angle (in °) | 77 [75-79] | 77 [75-77] | Ns |
| Li30 (in %) | 100 [100-100] | 100 [100-100] | Ns |
| Li45 (in %) | 98 [96-99] | 97 [95-98] | Ns |
| Li60 (in %) | 94 [92-96]* | 93 [91-95] | Ns |
| **INTEM** | | | |
| CT (in s) | 172 [162-184] | 172 [164-179] | Ns |
| A5 (in mm) | 52 [48-56] | 52 [50-52] | Ns |
| A10 (in mm) | 61 [58-65] | 61 [59-61] | Ns |
| CFT (in s) | 59 [50-69] | 58 [50-64] | Ns |
| MCF (in mm) | 67 [64-70] | 66 [65-70] | Ns |
| Alpha angle (in °) | 78 [76-80] | 78 [77-79] | Ns |
| Li30 (in %) | 100 [99-100] | 100 [99-100] | Ns |
| Li45 (in %) | 97 [95-98] | 96 [95-96] | Ns |
| Li60 (in %) | 94 [91-96]* | 92 [91-94] | Ns |
| **FIBTEM** | | | |
| CT (in s) | 59 [54-64] | 58 [53-67] | Ns |
| A5 (in mm) | 18 [15-21] | 18 [17-20] | Ns |
| A10 (in mm) | 19 [16-23] | 19 [18-22] | Ns |
| MCF (in mm) | 20 [17-24] | 20 [19-24] | Ns |
| Li30 (in %) | 100 [100-100] | 100 [100-100] | Ns |
| Li45 (in %) | 100 [100-100] | 100 [100-100] | Ns |
| Li60 (in %) | 100 [99-100] | 100 [100-100] | Ns |
| **TPA** | | | |
| CT (in s) | 58 [47-74]* | 57 [47-62] | Ns |
| A5 (in mm) | 52 [47-57]* | 54 [50-55] | Ns |
| A10 (in mm) | 60 [54-65]* | 61 [60-64] | Ns |
| CFT (in s) | 60 [51-76]* | 55 [52-67] | Ns |
| MCF (in mm) | 64 [58-69]* | 64 [61-67] | Ns |
| Alpha angle (in °) | 78 [75-79]* | 79 [76-79] | Ns |
| Li30 (in %) | 97 [86-99]* | 96 [83-98] | Ns |
| Li45 (in %) | 61 [20-83]* | 55 [26-72] | Ns |
| Li60 (in %) | 7 [2-31]** | 2 [1-6] | Ns |
| LOT (in s) | 2261 [1836-2643]ᶲ | 2188 [1759-2418] | Ns |
| LT (in s) | 3433 [2868-4005]ᶲᶲ | 3266 [2763-3411]* | Ns |
| **Routine hemostasis assays** | | | |
| Fibrinogen (in g/L) | 3.6 [3.0-4.2]* | 3.6 [3.1-4.0]* | Ns |
| PT (in s) | 10.6 [10.3-10.9]* | 10.6 [10.3-11.8]* | Ns |
| aPTT (in s) | 26 [25-27]* | 27 [25-29]* | Ns |
| Platelets (in 10^9^/L) | 250 [214-292] | 232 [189-260] | 0.078 |

*Missing value for 1 patient; ** missing values for 2 patients; ᶲ missing values for 7 patients; ᶲᶲ missing values for 9 patients

CT: Clotting Time, CFT: Clot Formation Time, AX: Amplitude at X minutes, MCF: Maximum Clot Firmness, LiX: Lysis index at X minutes, LOT: Lysis Onset Time, LT: Lysis Time
